# Ctip2-, Satb2-, Prox1-, and GAD65-Expressing Neurons in Rat Cultures: Preponderance of Single- and Double-Positive Cells, and Cell Type-Specific Expression of Neuron-Specific Gene Family Members, Nsg-1 (NEEP21) and Nsg-2 (P19)

**DOI:** 10.1371/journal.pone.0140010

**Published:** 2015-10-14

**Authors:** Laura Digilio, Chan Choo Yap, Bettina Winckler

**Affiliations:** University of Virginia, Department of Neuroscience, 409 Lane Road, Charlottesville, VA, 22908, United States of America; University of Freiburg, GERMANY

## Abstract

The brain consists of many distinct neuronal cell types, but which cell types are present in widely used primary cultures of embryonic rodent brain is often not known. We characterized how abundantly four cell type markers (Ctip2, Satb2, Prox1, GAD65) were represented in cultured rat neurons, how easily neurons expressing different markers can be transfected with commonly used plasmids, and whether neuronal-enriched endosomal proteins Nsg-1 (NEEP21) and Nsg-2 (P19) are ubiquitously expressed in all types of cultured neurons. We found that cultured neurons stably maintain cell type identities that are reflective of cell types *in vivo*. This includes neurons maintaining simultaneous expression of two transcription factors, such as Ctip2+/Satb2+ or Prox1+/Ctip2+ double-positive cells, which have also been described *in vivo*. Secondly, we established the superior efficiency of CAG promoters for both Lipofectamine-mediated transfection as well as for electroporation. Thirdly, we discovered that Nsg-1 and Nsg-2 were not expressed equally in all neurons: whereas high levels of both Nsg-1 and Nsg-2 were found in Satb2-, Ctip2-, and GAD65-positive neurons, Prox1-positive neurons in hippocampal cultures expressed low levels of both. Our findings thus highlight the importance of identifying neuronal cell types for doing cell biology in cultured neurons: Keeping track of neuronal cell type might uncover effects in assays that might otherwise be masked by the mixture of responsive and non-responsive neurons in the dish.

## Introduction

It is well known that the brain consists of hundreds of distinct neuronal cell types [1–3] which express different proteins, including receptors, transcription factors, and adhesion molecules that lead to distinct developmental programs, distinct axon outgrowth decisions, distinct circuit formation, distinct excitability, and ultimately distinct functions in the circuits (for example [4,5]). Cell biologists interested in neuronal function at the single cell or subcellular level often use primary neurons in culture [6] because many cell biological assays do not yet lend themselves to study in the whole animal. Labs interested in synapses routinely distinguish broadly between inhibitory (GABAergic) and excitatory pyramidal type (glutamatergic) neurons in culture using well-established markers for inhibitory or excitatory pre- and postsynaptic proteins [7,8]. For studies of other cell biological problems (such as axon and dendrite specification and growth, in vitro guidance, polarized trafficking), the particular cell type of an individual cell studied is rarely known, and it is usually assumed that the basic cell biology is identical in all neuronal subtypes. Even though all neurons share many features of basic cell biological regulation, cell-type specific responses also exist. In beautiful work, the Benson lab showed that some, but not all, cultured cortical neurons respond to Sema3A by collapsing their growth cones. They discovered that the observed neuron-to-neuron variability was due to cell-type differences [9]. Sensitivity to Aβ toxicity is also different in distinct neuronal cell types in cultures [10]. The neuron-to-neuron variability frequently encountered in cellular assays might thus be due to different biology in distinct neuronal cell types.

We thus asked what neuronal cell types we can identify in hippocampal and cortical cultures derived from E18 rat embryos. The characterization of distinct neuronal cell type has made enormous strides in the last decade, especially by identifying transcription factors driving distinct neuronal fate decisions during development of the neocortex [2]. Work in the developing hippocampus has also led to a better understanding of how different hippocampal cell types are specified [11,12,13]. In this work, we characterized the preponderance of expression of four markers (Ctip2, Satb2, Prox1 and GAD-65) and the expression efficiencies of four promoters using two transfection methods commonly employed in cultured neurons (Lipofectamine and electroporation). Using quadruple immunofluorescence, we identified subpopulations of singly and dually positive neurons and found that different promoters show preferential expression in different cell types. Lastly, we used our new characterization of neuronal cell types in culture to ask if the neuronal enriched protein NEEP21/Nsg-1 and its related family member p19/Nsg-2 are expressed equally in all neurons in culture. Unexpectedly, NEEP21 and P19/Nsg-2 are barely expressed in Prox1-positive neurons. Our findings highlight the importance of tracking neuronal cell types and will provide useful tools to researchers facing neuron-to-neuron variability in culture.

## Results

### Do different markers commonly used to identify neurons stain the same cells in cultured neurons?

**Fig 1:**
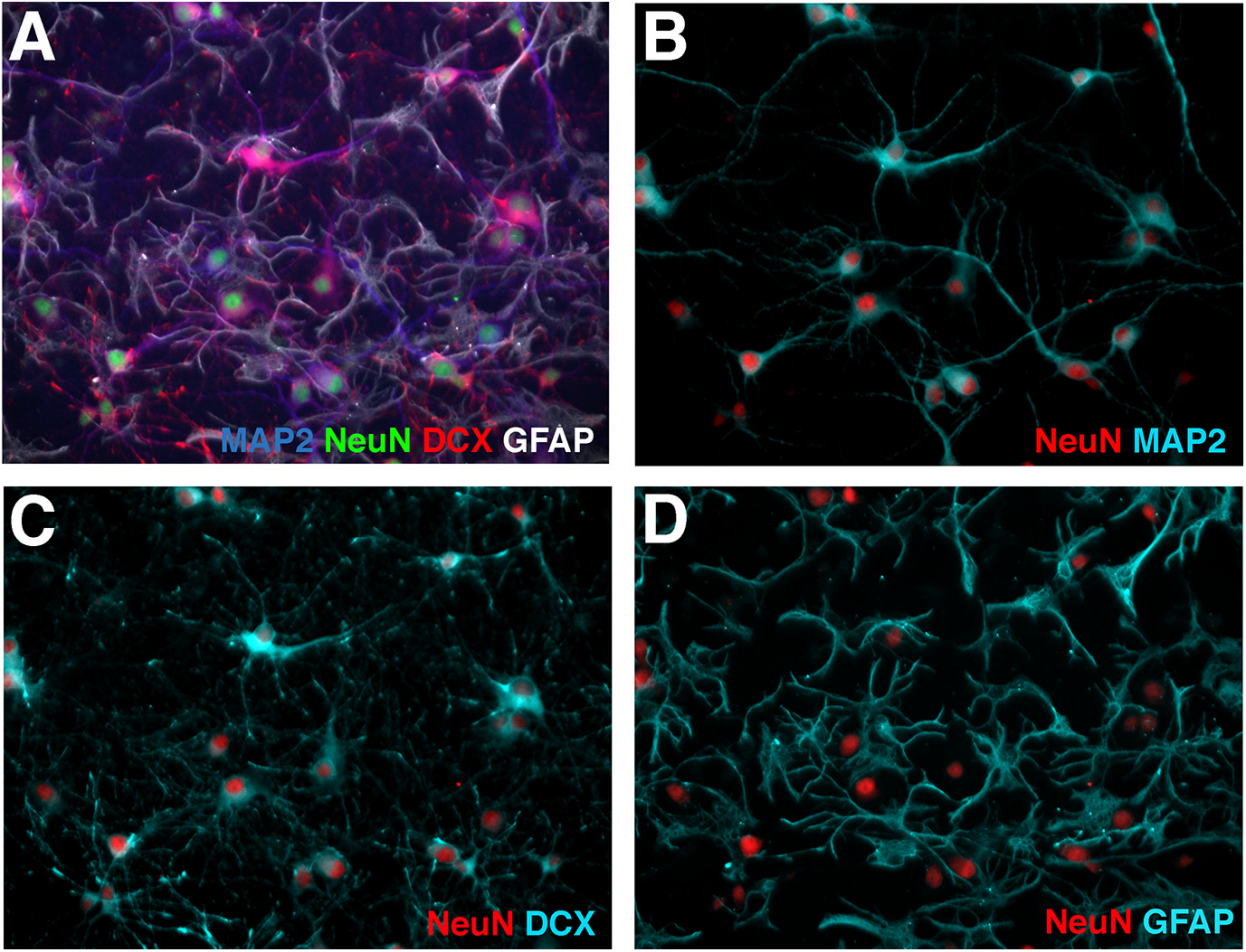
NeuN, MAP2, and DCX interchangeably identify neurons in DIV9 rat hippocampal neurons. (A) Quadruple staining of DIV9 rat hippocampal cultures with MAP2 (blue), DCX (red), NeuN (green), and GFAP (white). (B-D) NeuN (red) is shown in double combinations with each of the other markers (aqua) from panel (A) for easier viewing. (B) NeuN with MAP2, (C) NeuN with DCX (D) NeuN with GFAP. 95% of NeuN-positive cells also express MAP2 and DCX.

We began our analysis in hippocampal cultures grown for nine days (9 days in vitro = DIV9) by testing the co-expression of three markers for neurons: MAP2, DCX (doublecortin) and NeuN. These markers are widely used to distinguish neuronal from non-neuronal cells in brain sections and in cultures. MAP2 is a cytoskeletal protein, which preferentially decorates dendrites in neurons [14]. DCX is expressed highly in developing neurons and is considered to be restricted to neuroblasts (which can divide once more) and to postmitotic newborn neurons [15–17]. DCX expression is downregulated later in development. NeuN is a transcription factor expressed in most mature neurons, but is not detected in newborn neurons. As a control, we also stained for GFAP, a marker of astrocytes. Astrocytes are present in our cultures since we purposefully do not add anti-mitotic agents, such as AraC. We find that addition of AraC leads to less healthy neurons and lower survival after transfections. Since antibodies against these common markers are widely available commercially (see Table 1 in Materials and Methods), we optimized immunofluorescence staining using antibodies raised in different species (anti-MAP2 in chick, anti-GFAP in goat, anti-NeuN in guinea pig, anti-DCX in rabbit) to carry out quadruple immunostainings (Fig 1A).

GFAP-positive cells never overlapped with MAP2-, DCX-, or NeuN-positive cells. The co-staining of just the NeuN and GFAP channels are shown in Fig 1D. The three neuronal markers showed overwhelming co-localization (95%) (Fig 1B and 1C). The other 5% of cells were represented by a small proportion of NeuN-positive cells that were positive for DCX, but not for MAP2 (~1%) and by cells that expressed MAP2, but not any of the other markers NeuN, DCX, or GFAP (~4%). MAP2, NeuN, and DCX can thus be interchangeably used with 95% fidelity to identify neurons in DIV9 hippocampal cultures.

### Does the proportion of identifiable neuronal subtype change with days in vitro?

We next tested several cell-type markers often used by researchers to distinguish distinct cell types in brain sections. Antibodies against Satb2, Ctip2, GAD65, Prox1, Cux1, Pax6 and Tbr1 were tested, but only antibodies against Ctip2, Satb2, GAD65 and Prox1 gave satisfactory staining in DIV9 rat hippocampal cultures (see Materials and Methods, Table 1). Prox1 is a transcription factor expressed highly in dentate gyrus granule neurons, and genetic loss of Prox1 favors pyramidal cell fate [18]. Prox1 is also expressed in the CA region of the hippocampus, but only in a small subset of cells scattered throughout [19]. In cortex, Prox1 defines a subset of interneurons [20]. Satb2 and Ctip2 are transcription factors best studied for their roles in specifying callosal vs. subcortical projection neurons in the neocortex [21,22], respectively. mRNA expression of both transcription factors during embryogenesis is low in the hippocampus compared to the neocortex. Expression levels increase greatly with age, and expression in the hippocampus is robust postnatally. Ctip2 expression was first detected in E15 mouse hippocampus in the CA1 region. Expression expands to the developing dentate gyrus by E18 and continues to be strongly expressed postnatally in dentate granule neurons [13,23]. In fact, Ctip2 was shown to be required for postnatal dentate gyrus development [23]. Ctip2 remains low or not detectable in the CA3 region throughout development and into adulthood. Satb2 is expressed early in the CA1 region where it co-localizes with Ctip2 [13,24]. In situ hybridization data is available from the Allen Brain Institute public database and is shown in S1 Fig for the convenience of the reader.

**Fig 2:**
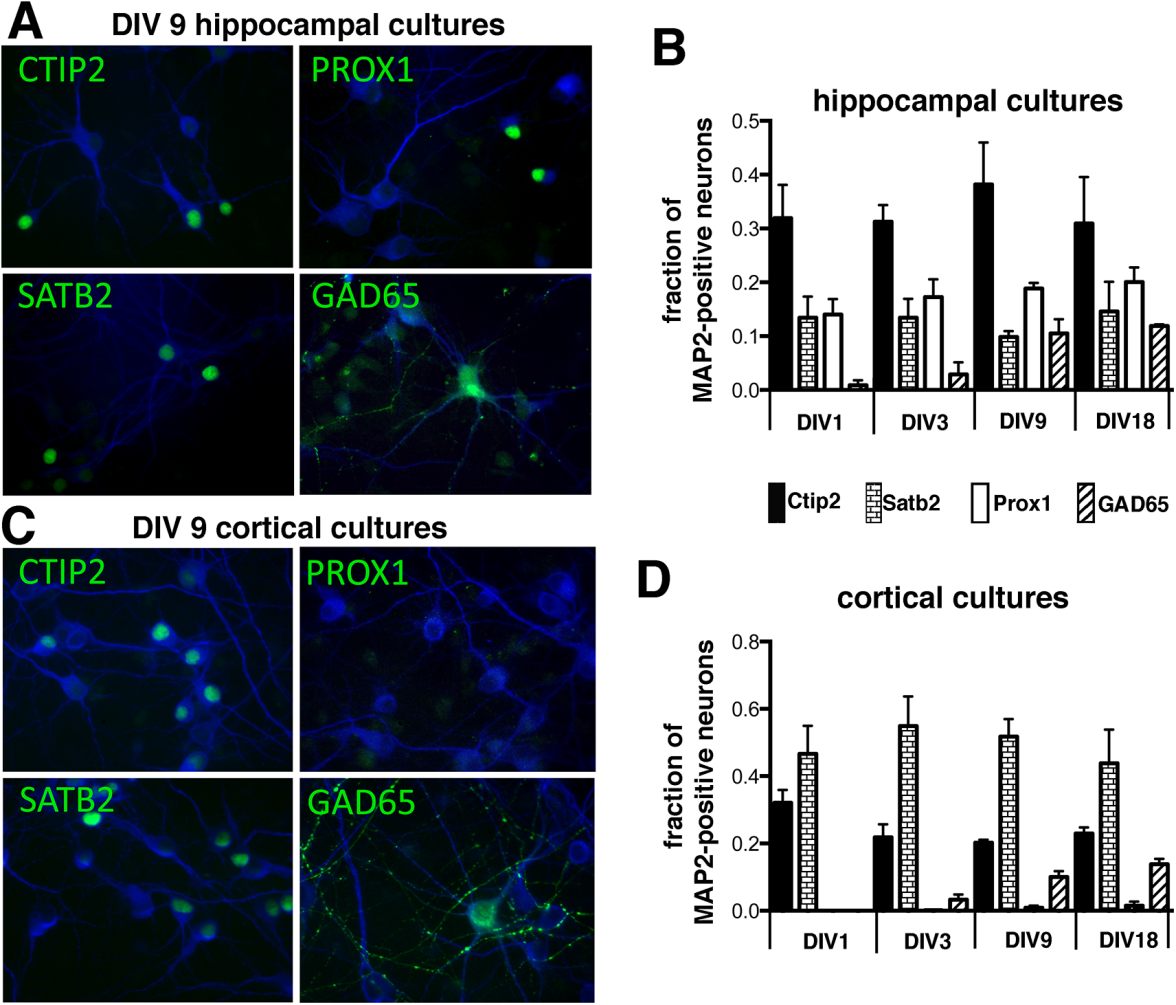
Time course of expression of Ctip2, Satb2, GAD65 in cultures derived from E18 rat hippocampus or cortex. (A) DIV9 hippocampal neurons were stained with MAP2 and counterstained against Ctip2, Prox1, Satb2, and GAD65. (B) E18 hippocampal cultures were stained at DIV1, 3, 9, 18 as in (A) and the proportion among MAP2-positive neurons was quantified in three separate experiments. Means and standard deviations are shown. Black bars = Ctip2-positive; brick bars = Satb2-positive; white bars = Prox1-positive; striped bars = GAD65-positive. In each experiment, 300 to >1000 MAP2-positive neurons were counted. (C) DIV9 cortical neurons were stained with MAP2 and counterstained against Ctip2, Prox1, Satb2, and GAD65. (D) E18 cortical cultures were stained at DIV1, 3, 9, 18 as in (C) and the proportion among MAP2-positive neurons was quantified in three separate experiments. Means and standard deviations are shown. Black bars = Ctip2-positive; brick bars = Satb2-positive; white bars = Prox1-positive; striped bars = GAD65-positive. In each experiment, 300 to >1000 MAP2-positive neurons were counted.

We characterized the preponderance of these markers in hippocampal cultures. Cultures were counterstained against MAP2, and the proportion of MAP2-positive neurons also positive for one of the markers was determined for three independent cultures. Examples of DIV9 cultures are shown in Fig 2A.

Ctip2-positive neurons are the most abundant (~38%), followed by Prox1-positive neurons (~20%), with GAD65- and Satb2-positive neurons each comprising ~10% of MAP2-positive neurons (Fig 2B). We then extended the marker analysis to additional time points in culture, starting at DIV1 and ending at DIV18. The relative preponderance was similar at all ages tested (Fig 2B) with the exception of GAD65. GAD-65-positive neurons increased in abundance with time in culture from barely detectable to ~12% of MAP2-positive neurons. GAD65 is thus a later marker for GABAergic neurons and is not expressed in early cultures. GAD65 can therefore only be used to identify GABAergic neurons in older cultures.

### What is the proportion of neuronal subtypes in cortical cultures?

We next used the same antibodies to characterize the preponderance of Ctip2, Satb2, Prox1, and GAD65 in DIV 9 cultures derived from E18 rat cortex (Fig 2C). Satb2-positive neurons were the most abundant (~50% of MAP2-positive neurons (blue)). Ctip2 accounted for 20–30% of MAP2-positive neurons. Prox1-positive neurons were virtually absent from cultures derived from E18 cortex. Quantification of additional time points in culture showed that, similarly to hippocampal cultures, the proportion of each neuronal cell type was quite stable between DIV1 and DIV18 (Fig 2D). Also as seen for hippocampal cultures, GAD65 preponderance increased with time in culture (Fig 2D). Cortical cultures derived from E18 rat cortex thus express markers expressed by neurons residing in layers II/III as well as in layer V.

### How many of the neurons can be identified using four markers for neuronal subtypes?

**Fig 3:**
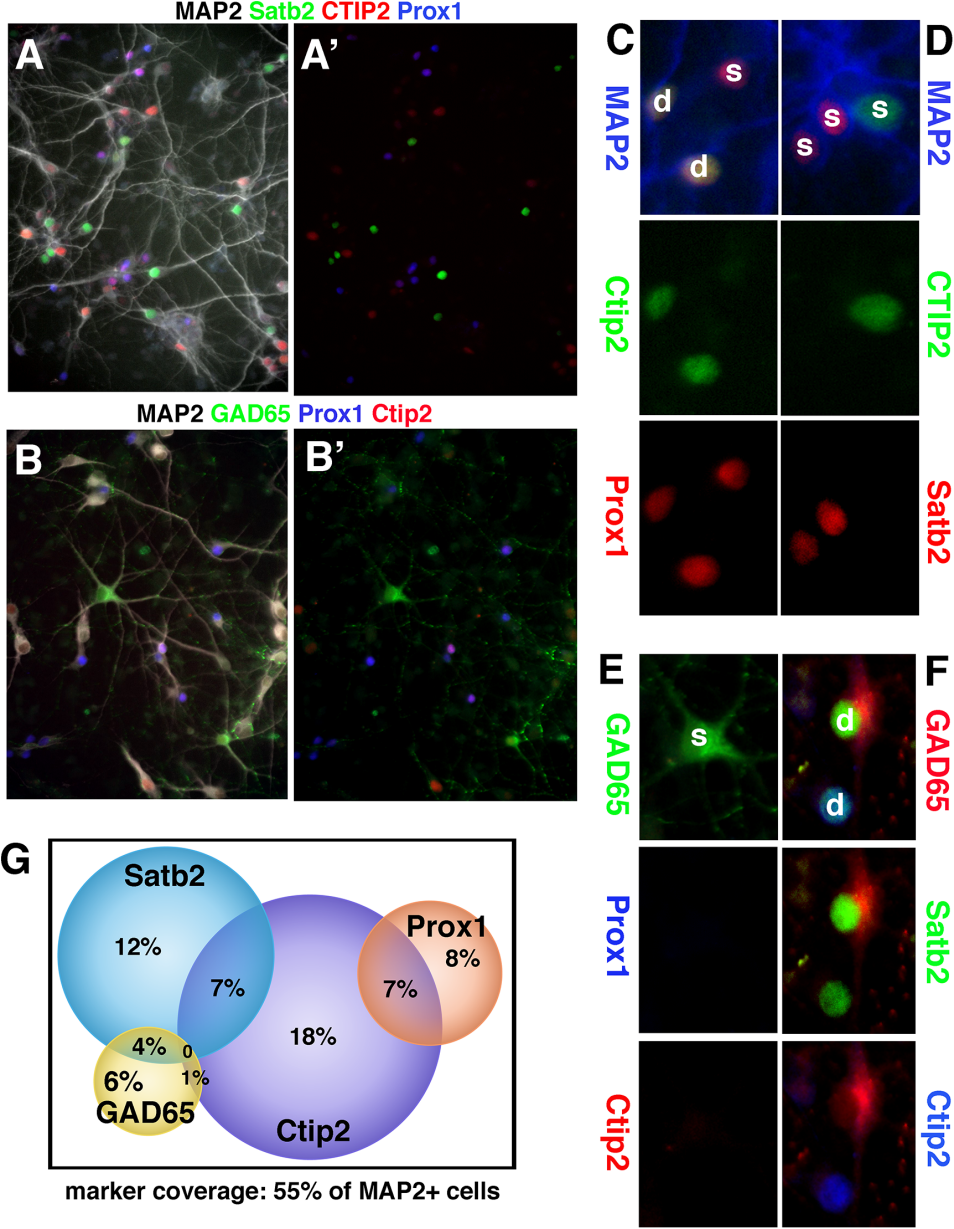
Non-exclusivity in expression of transcription factors used as cell type markers. (A,B) Quadruple stainings of DIV9 rat hippocampal neurons with MAP2 (white), Ctip2 (red), Satb2 (green), and Prox1 (blue) (A), or MAP2 (white), GAD65 (green), Prox1 (blue), Ctip2 (red) (B). (A’) and (B’) show the same field with MAP2 staining omitted. (C-F) show examples of single (s) and dually (d) positive neurons. (C) Ctip2/Prox1-single (s) and dually (d) positive neurons. (D) single-positive Ctip2- or Satb2-positive neurons. (E) Gad65-positive neuron not expressing Prox1 or Ctip2. (F) GAD65/Satb2 double-positive neuron and Ctip2/Satb2 double-positive neuron. (G) Venn diagram showing the abundance of single and double-positive neurons. Two independent DIV9 hippocampal cultures were scored and normalized to MAP2-positive cells. In each experiment, 300–400 MAP2-positive neurons were counted.

We then wondered what proportion of MAP2-positive neurons we could account for using the four markers described in Fig 2. If we assume that all four markers used were expressed in unique, non-overlapping sets of neurons, we can account for ~ 75% of neurons in DIV9 hippocampal cultures and ~ 85% of DIV9 cortical cultures. A straight accounting of cell types using MAP2 and one other marker is only accurate, though, if markers are expressed in a mutually exclusive manner. We thus wondered how many of the identified cell types might be positive for more than one of the cell type markers. We thus devised a staining protocol to stain simultaneously for MAP2 and three other markers in DIV9 hippocampal cultures. We used a chicken anti-MAP2 antibody in combination with two mouse antibodies and a rabbit antibody. Two mouse antibodies can be used in the same staining if the primary antibodies are first coupled to different fluorophore-carrying secondary antibodies using the Zenon labeling kit (see Materials and Methods for details). Controls were performed to make sure that Zenon-labeled mouse antibodies did not crosslabel (data not shown). Using the Zenon kit to stain with two mouse antibodies, we were able to stain the following three combinations: MAP2-Ctip2-Satb2-GAD65, MAP2-Ctip2-Satb2-Prox1, and MAP2-Prox1-Ctip2-GAD65 (Fig 3).

Four-color overlays (red-green-blue-white) for two of the combinations are shown in Fig 3A and 3B. In 3A’ and 3B’, the MAP2 channels were omitted to allow easier viewing of co-localization. The percentage of co-localization was counted for MAP2-positive neurons and data combined into a Venn diagram (Fig 3G). 19% of MAP2-positive neurons were dually positive with the markers used. Prox1/Ctip2 double-positive neurons and Ctip2/Satb2 double-positive neurons were the most abundant at 7% each. Of all Prox1-positive neuron (~15% of MAP2-positive cells), about half were double positive with Ctip2. An example is shown in Fig 3C where “s” marks single-positive neurons, and “d” marks double-positive neurons. Satb2 and Ctip2 often marked mutually exclusive cell populations (Fig 3D), but 22% (7% out of 32%) of Ctip2-positive neurons also expressed Satb2 (Fig 3F, lower cell marked “d”). Conversely, ~30% of Satb2-positive neurons also expressed Ctip2 (7% of ~23%). ~45% of GAD65-positive neurons (~11% of all MAP2-positive neurons) were single-positive (Fig 3E), but double-positive GAD65 neurons could be observed, most often with Satb2 (Fig 3F, upper cell marked with “d”). Taken the double-positive populations into account, we can thus account for ~55% of MAP2-positive neurons in DIV9 hippocampal cultures using just 4 subtype markers. Of these identifiable neuronal subtypes, about one third (19% out of 55%) are positive for two markers.

### Can Ctip2, Satb2, and Prox1 neuronal subtypes be transfected with similar efficiencies?

**Fig 4:**
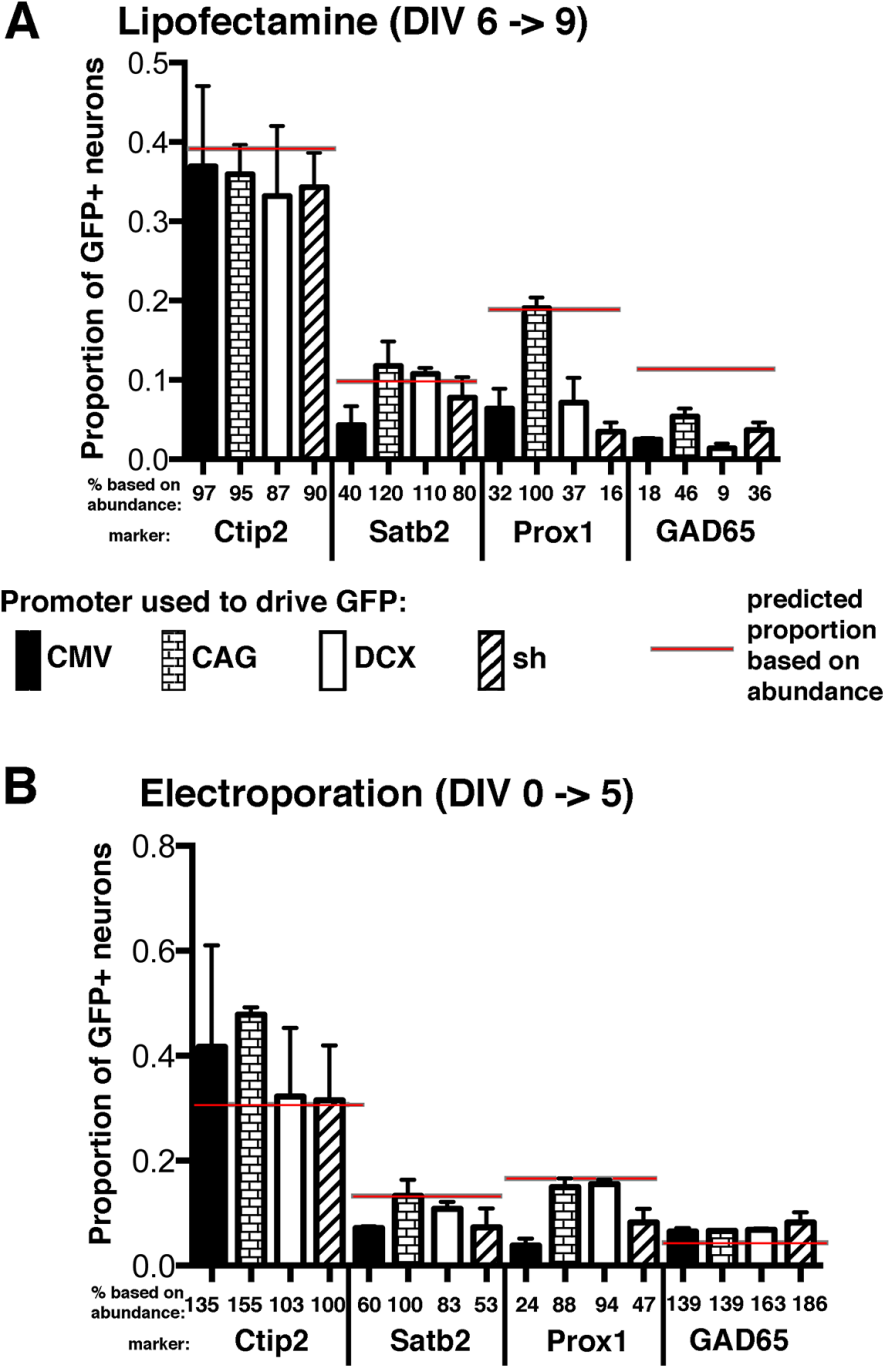
Differential transfectability of different neuronal cell types. Hippocampal neurons were either transfected with Lipofectamine2000 at DIV6 (A) or eletroporated at DIV0 prior to plating (B) and GFP-expressing MAP2-positive neurons were scored for the presence of Satb2, Ctip2, Prox1, or GAD65 (as indicated) at DIV9 (A) or DIV5 (B). The relative abundance of each marker-positive neuron is indicated by the red bar (data from Fig 2). Four GFP-encoding plasmids were used: CMV-promoter driven GFP (black bars), CAG-promoter driven (brick bars), DCX-promoter driven (white bars), short hairpin plasmid (sh; striped bars). The transfection efficiency normalized to the relative abundance is indicated as percentages below each bar. The mean and standard deviation of 2 independent experiments is shown. Total number of MAP2-positive neurons counted was between 75 and >400 per marker and experiment, depending on the abundance of the marker.

Many labs routinely transfect cultured neurons for experiments, but which neuronal cell types are transfected is not known. We thus asked if transfection method or choice of promoter affected the efficiency with which different neuronal cell types could be transfected in culture. We chose two transfection methods, which we rely on heavily in our own experiments [25,26], Lipofectamine2000 at DIV6-9 and electroporation prior to plating. We first tested a GFP-encoding plasmid driven by the popular CMV promoter. Surprisingly, we found that CMV-promoter driven GFP is not evenly represented among the identifiable cell types. Ctip2-positive cells transfected with CMV-GFP are recovered at their representative ~38% abundance (97% of predicted; black bar in Fig 4A), but Prox1-, Satb2- and GAD65-positive cells are greatly underrepresented among the CMV-GFP expressing cells (black bars in Fig 4A):

Satb2-positive neurons are transfected with CMV-GFP at 40% of predicted frequency, Prox1-positive neurons at 32% of predicted frequency, and GAD65-positive neurons at 18% of predicted frequency. Using electroporation of freshly dissociated cells to introduce CMV-GFP also shows differential transfectability in different cell types. Again Ctip2-positive cells show percentages representative of their overall abundance in the culture (black bar in Fig 4B) whereas Satb2-positive and Prox1-positive GFP-expressing neurons are underrepresented, 60% and 24% respectively. In contrast to Lipofectamine, GAD65-positive neurons expressed GFP after electroporation with CMV-GFP at the predicted abundance (Fig 4B).

We then tested three other promoters, the actin enhancer in combination with CMV (“CAG”), which is popular for high expression in neurons for in utero electroporations, a minimal DCX promoter, and the RNA Polymerase II-based U1 promoter frequently used for driving expression of short hairpin (sh) constructs. The CAG promoter was by far the most efficient in driving proportional representation in different cell types (Fig 4A and 4B brick bars), with the exception of expression in GAD65-positive neurons after Lipofectamine which was only 46% of predicted frequency (Fig 4A). Using Lipofectamine transfections, the DCX promoter was efficiently expressed in Ctip2-, and Satb2-positive cells (87% and 110% of predicted frequency, respectively), but DCX-GFP was underrepresented in Prox1- and GAD65-positive cells 37% and 9% of predicted frequency). After electroporation, DCX-GFP was expressed proportionally to abundance in all subcategories of cell types (white bars in Fig 4B). This probably reflects the younger age of the cultures where the DCX promoter is still active at higher levels across all cell types. The short hairpin sh-promoter was most comparable to the CMV promoter and was mostly underrepresented, except in Ctip2-positive neurons and in GAD65-positive neurons after electroporation (striped bars in Fig 4).

### Are the neuronal-specific endosomal proteins Nsg-1/NEEP21 and P19/Nsg-2 expressed widely in Ctip2-, Satb2-, and Prox-1 positive neurons?

**Fig 5:**
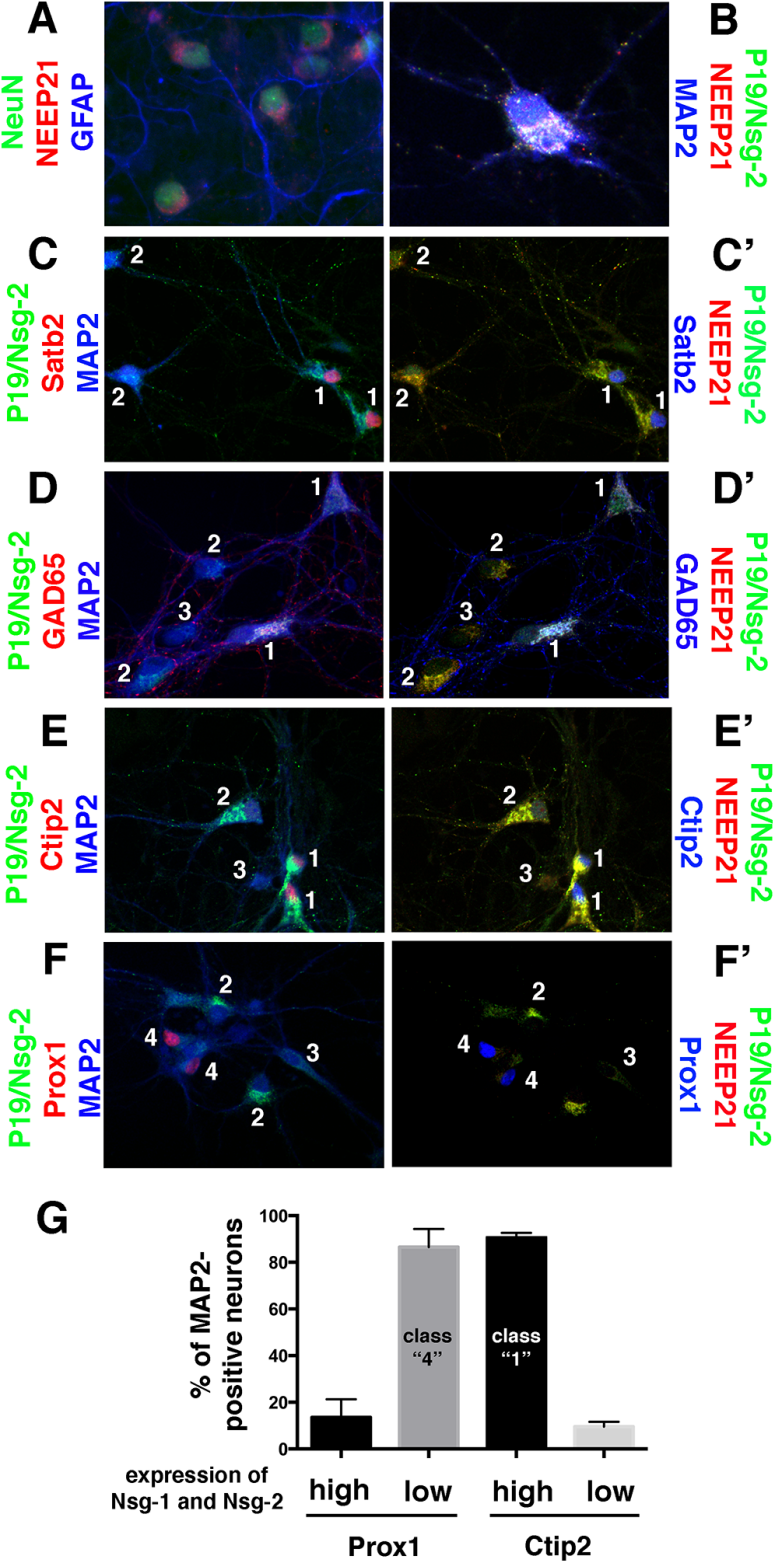
Neuronally enriched endosomal proteins Nsg-1 (NEEP21) and Nsg-2 (P19) are highly expressed in Satb2-, Ctip2-, and GAD65-positive neurons, but expressed at low/undetectable levels specifically in Prox1-positive neurons. (A) Nsg-1/NEEP21 (red) is specifically enriched in Neu-N-positive neurons (green), but undetectable in GFAP-positive astrocytoes (blue) in DIV9 rat hippocampal cultures. (B) Nsg-1/NEEP21 (red) and its family member Nsg-2/P19 (green) are present in the somatic Golgi region as well as in dispersed dendritic endosomes. (C-G) Nsg-1/NEEP21 and Nsg-2/P19 were co-localized with cell-type markers Satb2 (C, C’), GAD65 (D, D’), Ctip2 (E, E’), and Prox1 (F, F’). For each combination, the cell type marker is shown with either MAP2 (blue) and P19/Nsg-2 (green) (C, D, E, F), or with Nsg-1/NEEP21 (red) and Nsg-2/P19 (green) (C’,D’, E’, F’). In each panel, cells positive for the marker and NEEP21+P19 are labeled with “1”, cells negative for the marker and positive for NEEP21+P19 with “2”, cells negative for the marker and low for NEEP21+P19 with “3”, and cells positive for the marker but low for NEEP21+P19 with “4”. (G) Quantification of MAP2-positive neurons positive for one marker (Ctip2, Prox1) expressing either high levels of Nsg-1/NEEP21 and Nsg-2/P19 (black bars; correspond to type “1” neurons in panels C-F) or low levels of NEEP21+P19 (grey bars; correspond to type “4” neurons in panels C-F). Several hundred MAP2-positive neurons were scored for each marker in each of two independent experiments.

Neurons overall share many pathways and molecular players with non-neuronal cells, but work from our lab and others is also illuminating to what extent neurons utilize distinct machinery (for example [25,27–32]. Our lab is particularly interested in regulation of endosomes, and we have in the past described both neuronal-specific roles for ubiquitous endosomal regulators [33] as well as roles of neuronal-enriched endosomal proteins, such as NEEP21 (aka Nsg-1) [25,34]. Fig 5A shows the neuronal expression of Nsg-1/NEEP21, which co-expressed in NeuN-positive cells but was not detectable in GFAP-positive astrocytes in cultured hippocampal neurons.

Nsg-1/NEEP21 localization to the neuronal Golgi region and to dendritic endosomes has been previously reported [27,34,35]. Localization to the Golgi was also reported for the related family member Nsg-2/P19 [36]. In order to determine if Nsg-1/NEEP21 and Nsg-2/P19 co-localized in neurons, we carried out simultaneous immunofluorescence against Nsg-1/NEEP21 and Nsg-2/P19 together with MAP2 (Fig 5B). Nsg-1/NEEP21 and Nsg-2/P19 co-localized to Golgi/trans-Golgi network regions in the cell soma as well as to many endosomes dispersed along dendrites, but are largely excluded from the axon.

We previously noticed that some neurons appeared to express very little NEEP21. We thus asked if neuronal-enriched proteins, such as Nsg-1/NEEP21 and its family member Nsg-2/P19 [37], were expressed equally across all MAP2-positive neurons in rat hippocampal cultures by carrying out quadruple immunofluorescence against MAP2, Nsg-1/NEEP21, Nsg-2/P19 and one cell type marker (Fig 5C–5G). Nsg-1/NEEP21 and Nsg-2/P19 were either both highly expressed or both expressed at low levels. Satb2- (Fig 5C) and GAD65-positive neurons (Fig 5D) always expressed both Nsg-1/NEEP21 and Nsg-2/P19 highly. For Ctip2-positive and Prox1-positive neurons, we observed more heterogeneity of expression of Nsg-1/NEEP21 and Nsg-2/P19 (Fig 5E and 5F). We thus carried out quantification of Ctip2- and Prox1-positive neurons expressing Nsg-1/NEEP21 and Nsg-2/P19 (Fig 5G). Whereas ~90% of Ctip2-positive cells expressed high levels of both Nsg-1/NEEP21 and Nsg-2/P19, Prox1-positive neurons showed the reverse pattern: ~85% of Prox1-positive neurons contained only low levels of detectable staining against both Nsg-1/NEEP21 and Nsg-2/P19 (Fig 5G). We thus discovered a cell-type preferential expression of the neuronally-enriched endosomal proteins, Nsg-1 and Nsg-2.

## Discussion

In this work, we first investigated which neuronal marker was best to reliably stain neurons in DIV9 hippocampal cultures. We expected that DCX would be expressed less abundantly than the later NeuN marker. Surprisingly, NeuN, MAP2, and DCX are all co-expressed in 95% of cells and can thus be used equally well to counterstain neurons at DIV9 (Fig 1). Secondly, the cell type distribution we found in cultured neurons reflects *in vivo* observations of cell type preponderance with respect to Ctip2, Satb2, and Prox1. As *in vivo*, Prox1 is much more abundantly expressed in neurons derived from hippocampus as compared to cortex (Fig 2). Furthermore, the preponderance of any given marker is stable in cultures between DIV1 and DIV18 for both cortex and hippocampus, arguing that there is no preferential loss of cell types or loss of markers during this culture time frame. GAD65 was the one exception whose prevalence increased with time of culture.

Much to our surprise, we frequently found neurons double-positive for Satb2 and Ctip2 (Fig 3), markers shown to suppress each other’s expression and thus routinely used as marking mutually exclusive populations in the cortex [21]. Scouring the literature revealed that such double-positive cells are visible in published images [38] and have recently been explicitly described *in vivo*, for both the cortex and hippocampus [13,39]. Prox1/Ctip2 double-positive cells in the hippocampus have also recently been described *in vivo* during development [40]. We did not observe Prox1-positive cells co-expressing GAD65, even though Prox1-positive interneurons have been described in the cortex [20], transiently in the developing hippocampus [41], and in adult neurogenesis in the hippocampus [42]. The true complement of transcription factors in any given cell type is not yet known, but caution should be exercised for designating a cell type based on a single transcription factor. As more transcription factors are identified and better antibodies become available, the co-expression should be revisited *in vivo* as well. For instance, we could not identify papers in the literature that GAD65 is co-expressed with Satb2 *in vivo*, a combination we did observe in culture. Satb2-positive GABAergic neurons can be generated from stem cells *in vitro* [43], but whether this occurs also *in vivo* remains an open question.

Our staining suggests that we have good representation of layer V neurons (Ctip2+) in E18 cortical cultures. Since Satb2 is widely expressed (layers II/III and layers IV/V), Satb2+ neurons in cortical cultures could be derived from any of these layers. For hippocampus, Prox1 is highly expressed in granule neurons in the dentate gyrus, as is Ctip2. Prox1-positive and Prox1+/Ctip2+ double-positive neurons in hippocampal cultures are thus likely derived from the dentate gyrus. Satb2+/Ctip2+ double-positive neurons are found in the CA1 region and are clearly present in our cultures. Ctip2 single-positive neurons likewise are likely derived from the CA1 region. Additional markers are still needed to completely account for all MAP2-positive neurons in hippocampal cultures. We were unable to see positive staining with antibodies against Cux2 and Tbr1, but this might be due to antibody incompatibility rather than non-expression in the cultures.

We also found unexpected differences in the transfection efficiencies of neuronal cell types with different promoters (Fig 4). The popular CMV-promoter drove GFP expression efficiently only in Ctip2-positive neurons, but Satb2-, Prox1- and GAD65-positive neurons were greatly underrepresented, especially when using Lipofectamine2000. The CAG-promoter drove expression much more efficiently in Satb2- and Prox1-positive neurons compared to the CMV-promoter. The DCX-promoter drove efficient expression especially after electroporation of freshly dissociated neurons. Investigators might thus choose a different promoter depending on their experimental question to either restrict or expand transfected cell types.

Lastly, we made the surprising discovery that Prox1-positive neurons (presumably corresponding to dentate gyrus-derived granule neurons) showed low abundance of the neuronal-specific proteins Nsg-1/NEEP21 and Nsg-2/P19 (Fig 5). Nsg-1/NEEP21 is a small transmembrane protein present in somatodendritic endosomes and has been implicated in regulating recycling of several neuronal proteins, including the L1 cell adhesion molecule [34], AMPA receptors [44], amyloid precursor protein [45], and neurotensin receptors [46]. When NEEP21 is downregulated, trafficking to LAMP1-positive lysosomes is increased. The cell type-specific low expression of Nsg-1/NEEP21 and Nsg-2/P19 raises the intriguing question of what the functional consequences are of differential expression of this class of endosomal proteins. It is possible that certain receptors might be differentially recycled or degraded by Prox1-positive neurons, leading to functional differences in axon guidance or synapse function. Answering this question is an exciting avenue of future research.

## Materials and Methods

### Antibodies

#### 1) Table 1: Commercially available primary antibodies

**Table 1:** Commercially available primary antibodies.

Antibody	Company	Catalog Number	Dilution
Chicken anti-MAP2	EnCor Biotechnology	CPCA-MAP2	1 to 2000
Rabbit anti-DCX	Santa Cruz Biotechnology	sc-8066	1 to 400
Goat anti-GFAP	Santa Cruz Biotechnology	sc-6170	1 to 400
Guinea Pig anti-Neun	Millipore	ABN90	1 to 1000
Mouse anti-SATB2	Abcam	ab51502	1 to 400
Rabbit anti-PROX1	Millipore	AB5475	1 to 1000
Mouse anti-PROX1	Active Motif	61093	1 to 1000
Mouse ant-GAD65	Millipore	MAB351	1 to 500
Rabbit anti-CTIP2	Novus Biologicals	NB100-2600	1 to 400
Rat anti-CTIP2	Biolegend	650601	1 to 400
Goat anti-NEEP21	Everest Biotech	EB11637	1 to 400

#### 2) Antibodies raised for this study

Polyclonal antibody against Nsg-2/P19 was raised in rabbits using a peptide analogous to the one described in [36](residues 138–155: HYSVAKQSTARAIGPWSL).

#### 3) Secondary antibodies

From Jackson Immunoresearch: Donkey anti-Chicken AMCA, Donkey anti-Guinea Pig Rhodamine, Donkey anti-Rat Alexa-647.

From Life Technologies: Donkey anti-Rabbit Alexa Fluor-488, -568, -647; Donkey anti-Mouse Alexa Fluor-488, -568, -647; Donkey anti-Goat Alexa Fluor-647.

All secondary antibodies were used at a 1:400 dilution.

### Plasmids

The following GFP-expressing plasmids were used: pCMV-EGFP-N3 (Clontech), pCAG-EGFP (Addgene, originally developed by C. Cepko), pDCX-IRES-EGFP (kindly provided by Frank Polleux, Columbia University; [47], pU1-shRandom-hMGFP (Qiagen).

### Preparation of embryonic rat cultures

Cultures were prepared as described previously [33]. Briefly, E18 timed pregnant Sprague Dawley females were euthanized using the approved CO_2_ chamber at the institutional animal facility. The University of Virginia IACUC approved this research (animal protocol #3422). The embryos were recovered in Hanks buffered salt solution (HBSS). The hippocampi and cortices were isolated separately and then incubated with a 1:10 dilution of trypsin in 5ml of HBSS at 37°C for 18 min. Following the incubation, the tissue was washed three times with HBSS and then resuspended in 2 ml of DMEM. The tissue was triturated gently 15x with a glass polished Pasteur pipet and then triturated again 15 times with a glass polished Pasteur pipet with a smaller diameter. Cells were counted and plated on poly-L-lysine coated coverslips and incubated in DMEM medium containing 10% horse serum. After 4h, the cells were transferred into serum-free medium supplemented with B27 (GIBCO BRL) and cultured for 1–18 DIV (days in vitro). Alternatively, neurons were electroporated after dissociation and then cultured for 5 days.

### Expression of GFP

**1) Transfection with Lipofectamine.** Hippocampal neurons were transfected with DNA plasmids using Lipofectamine 2000 as described [34] at DIV6 and incubated for 3 days, followed by fixing the cells at DIV9.

**2) Electroporation.** Freshly prepared dissociated neurons were electroporated using BTX 830 (Harvard Apparatus) following the manufacturer’s protocol. Briefly, about 700,000 neurons per transfection were suspended in 100μl of electroporation buffer (from BTX) together with 3-5ug of DNA, and electroporated with the following settings: 1^st^ pulse at 140V for 900μs followed by 2^nd^ pulse at 340V for 900μs. The electroporated neurons plated onto prepared coverslips coated with polylysine and were cultured for 5 days and fixed.

### Immunofluorescence protocol

**1) Fixation and immunostaining.** Cells were fixed in 2% paraformaldehyde/3% sucrose/PBS in 50% conditioned medium at room temperature for 30 minutes, washed and permeabilized with 0.2% Triton X-100 for 1h at room temperature. Cells were incubated with primary antibody diluted in PBS/1% BSA for 1h at RT. Cells were then washed and incubated with secondary antibodies diluted in PBS/1%BSA for 1 h at RT, washed and then mounted in Vectashield mounting media (Vector Labs).

**2) Quadruple staining of DCX, MAP2, NeuN and GFAP.** Antibodies raised in different species (chicken, rabbit, goat, guinea pig) were used to carry out quadruple staining. The antibodies used and dilutions are listed in Table 1.

**3) Quadruple staining of MAP2-Ctip2-Satb2-GAD65, MAP2-Ctip2-Satb2-Prox1, and MAP2- Prox1-Ctip2-GAD65 using Zenon kit.** For the quadruple staining of the markers, the Zenon (Life Technologies) antibody labeling system was used. Briefly, the combinations that were Zenon labeled together were GAD65 with Satb2, GAD65 with Prox1, and Satb2 with Prox1 using mouse monoclonal antibodies. The cells were fixed and permeabilized as above; the Zenon complexes of the mouse antibodies were prepared separately according to the protocol and then incubated with the cells for 1h at RT. Cells were then washed and fixed for 15 min with 4% paraformaldehyde, washed and then subsequently stained with the other antibodies (raised in chicken and rabbit) for 1h RT in PBS/1%BSA. Cells were washed with PBS and then incubated with secondary antibody (anti-rabbit and anti-chicken) diluted in PBS/1%BSA for 1h. Coverslips were washed and mounted with Vectashield mounting media.

**4) Nsg-2/P19 and Nsg-1/NEEP21 co-staining with markers.** For the Nsg-2/P19 and Nsg-1/NEEP21 staining, the cells were prewashed for 10 seconds with 0.02% saponin in culture media and then fixed as above. All antibody incubations were done in PBS/1%BSA/0.02% saponin for this antibody combination.

### Quantification

Cells were examined and imaged on a Zeiss Axiovert 200 microscope using either a 20x or 40x objective. For Fig 5, images were taken on a Zeiss880 laser scanning confocal (40x objective) using sequential acquisition of the red and green channels in order to maximally resolve the small endosomes. MAP2-positive cells were scored as positive or negative for the marker of interest; the percentage of positively stained cells was determined by counting all MAP2-positive cells as positive or negative for the marker of interest in a 20X or 40X field across the diameter of the coverslip. For quantification of quadruple labeled cell types, 20X or 40X images were captured with the Orca cooled CCD camera (Hamamatsu) using Openlab software (ImproVision) and then processed and scored in ImageJ, since scoring could not be done easily on the microscope and was thus done from merged images of all channels. Between 75 - >400 MAP2-positive cells were counted per marker in each experiment. Higher numbers of MAP2-positive cells were counted for less abundant marker (such as GAD65) to obtain reliable percentages. All counts were repeated on 2–3 independent cultures. Data was entered into Prism for graphing and error bars.

## Supporting Information

S1 Fig In situ hybridization of E18.5 mouse saggital sections according to Allen Brain Atlas (http://developingmouse.brain-map.org). (A) Schematized brain region map of E18.5 mouse brain saggital sections. The dorsal pallium (future neocortex) and ventral pallium (future hippocampus) are indicated by arrows for easier comparison to in situ panels shown in (B-E). (B-E) In situ hybridizations (left panels), and heat maps of regional abundance over developmental time (right panels) are shown for Gad2 (B), Ctip2 (C), Satb2 (D), and Prox1 (E). Black arrows point at the developing hippocampus, grey arrows point at the developing neocortex. E18 in situ hybridizations against rat brain could not be found in the database.
(TIF)
