# Supplementary figures and images for: Ctip2-, Satb2-, Prox1-, and GAD65-Expressing Neurons in Rat Cultures: Preponderance of Single- and Double-Positive Cells, and Cell Type-Specific Expression of Neuron-Specific Gene Family Members, Nsg-1 (NEEP21) and Nsg-2 (P19)
Source: PLoS One. 2015 Oct 14;10(10):e0140010. doi: 10.1371/journal.pone.0140010 (PMC4605768; doi:10.1371/journal.pone.0140010)

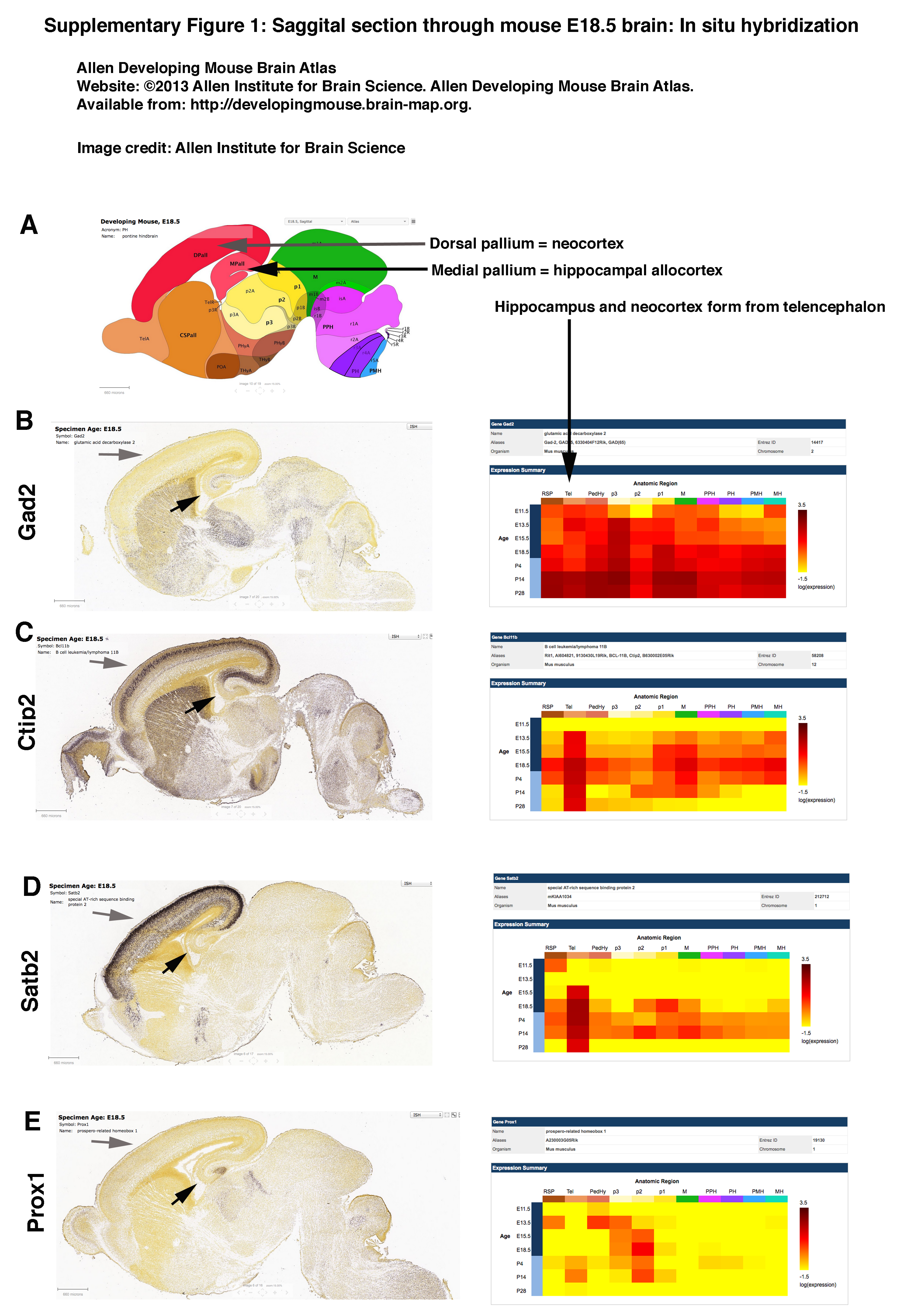

Supplement: S1 Fig — (A) Schematized brain region map of E18.5 mouse brain saggital sections. The dorsal pallium (future neocortex) and ventral pallium (future hippocampus) are indicated by arrows for easier comparison to in situ panels shown in (B-E). (B-E) In situ hybridizations (left panels), and heat maps of regional abundance over developmental time (right panels) are shown for Gad2 (B), Ctip2 (C), Satb2 (D), and Prox1 (E). Black arrows point at the developing hippocampus, grey arrows point at the developing neocortex. E18 in situ hybridizations against rat brain could not be found in the database. (TIF) [file pone.0140010.s001.tif]
